# Operative air temperature data for different measures applied on a building envelope in warm climate

**DOI:** 10.1016/j.dib.2018.02.030

**Published:** 2018-02-21

**Authors:** Cristina Baglivo, Paolo Maria Congedo

**Affiliations:** Department of Engineering for Innovation, University of Salento, 73100 Lecce, Italy

## Abstract

Several technical combinations have been evaluated in order to design high energy performance buildings for the warm climate. The analysis has been developed in several steps, avoiding the use of HVAC systems.

The methodological approach of this study is based on a sequential search technique and it is shown on the paper entitled “Envelope Design Optimization by Thermal Modeling of a Building in a Warm Climate” [1].

The Operative Air Temperature trends (TOP), for each combination, have been plotted through a dynamic simulation performed using the software TRNSYS 17 (a transient system simulation program, University of Wisconsin, Solar Energy Laboratory, USA, 2010).

Starting from the simplest building configuration consisting of 9 rooms (equal-sized modules of 5 × 5 m^2^), the different building components are sequentially evaluated until the envelope design is optimized. The aim of this study is to perform a step-by-step simulation, simplifying as much as possible the model without making additional variables that can modify their performances. Walls, slab-on-ground floor, roof, shading and windows are among the simulated building components. The results are shown for each combination and evaluated for Brindisi, a city in southern Italy having 1083 degrees day, belonging to the national climatic zone C. The data show the trends of the TOP for each measure applied in the case study for a total of 17 combinations divided into eight steps.

**Specifications Table**

**Table:** 

Subject area	*Civil engineering,*
More specific subject area	Design of high performance envelope for warm climate, considering the operative air temperature.
Type of data	*Tables*
How data was acquired	*Technical data sheet, analysed and processed output data*
Data format	*The data, presented in xls format, have been generated by the software TRNSYS 17. The building model has been designed through TRNBuild, an add-on of TRNSYS 17.*
Experimental factors	*The building material properties have been provided by commercial data sheets.*
Experimental features	*Each combination is characterized in terms of TOP.*
Data source location	*The evaluations have been done considering the temperature data of Brindisi, a city in southern Italy.*
Data accessibility	*Data is within this article.*

**Value of the data**

• The data show several high efficiency combinations for warm climate.
• It is useful for a further comparison with other studies conducted for other climates and for different uses.
• It facilitates the designer in the choice of interventions for highly efficient envelope, giving direct feedback about the variation of the operative air temperature trend.

## 1 Data

**Fig. 1:**
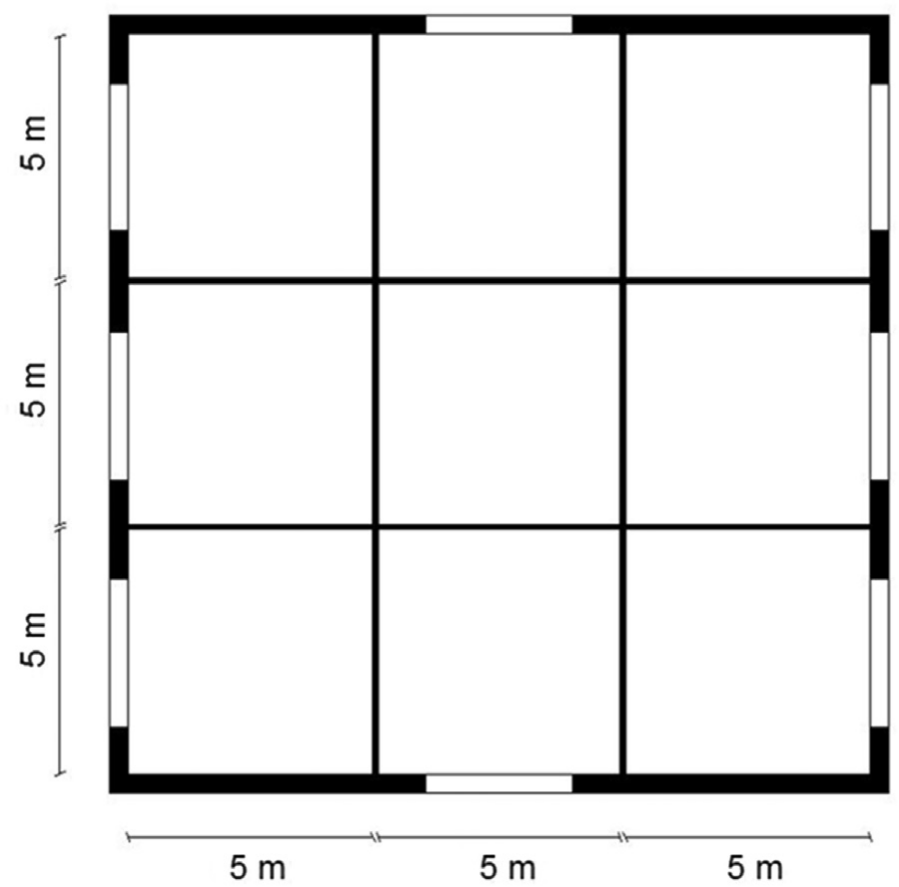
Case study plan.

The data are listed in the File “TOP in warm climate.xls”. The simulations have been performed by the use of the software TRNSYS 17. The case study is a hypothetical building prototype located in a warm climate. It is characterized by a square plan with a net surface area of 225 m^2^ and a net height of 2.7 m. The internal surface is divided into nine equal-sized modules of 5×5 m^2^, each module is a different thermal zone with a different orientation (Fig. 1).

The worksheet "Combo" reports the list of combinations for each step and shows the peak values for the maximum and minimum operative temperature obtained in summer and winter season, respectively. The external temperature and the internal gains are presented in the worksheet called “boundary conditions”. The “external walls” worksheet reports the characteristics of the wall building materials and the thermal properties of the five modelled walls (W1, W2, W3, W4 and W5). The thermo-physical properties of the selected options of slab-on-ground-floor are presented in the “ground floor” worksheet. Windows and roof properties are shown in the worksheets “windows” and “roof”.

The obtained values of TOP are exposed for each combination considering all orientations in the worksheets from "Combo 1" to "Combo 17".

## 2 Experimental design, materials and methods

**Fig. 2:**
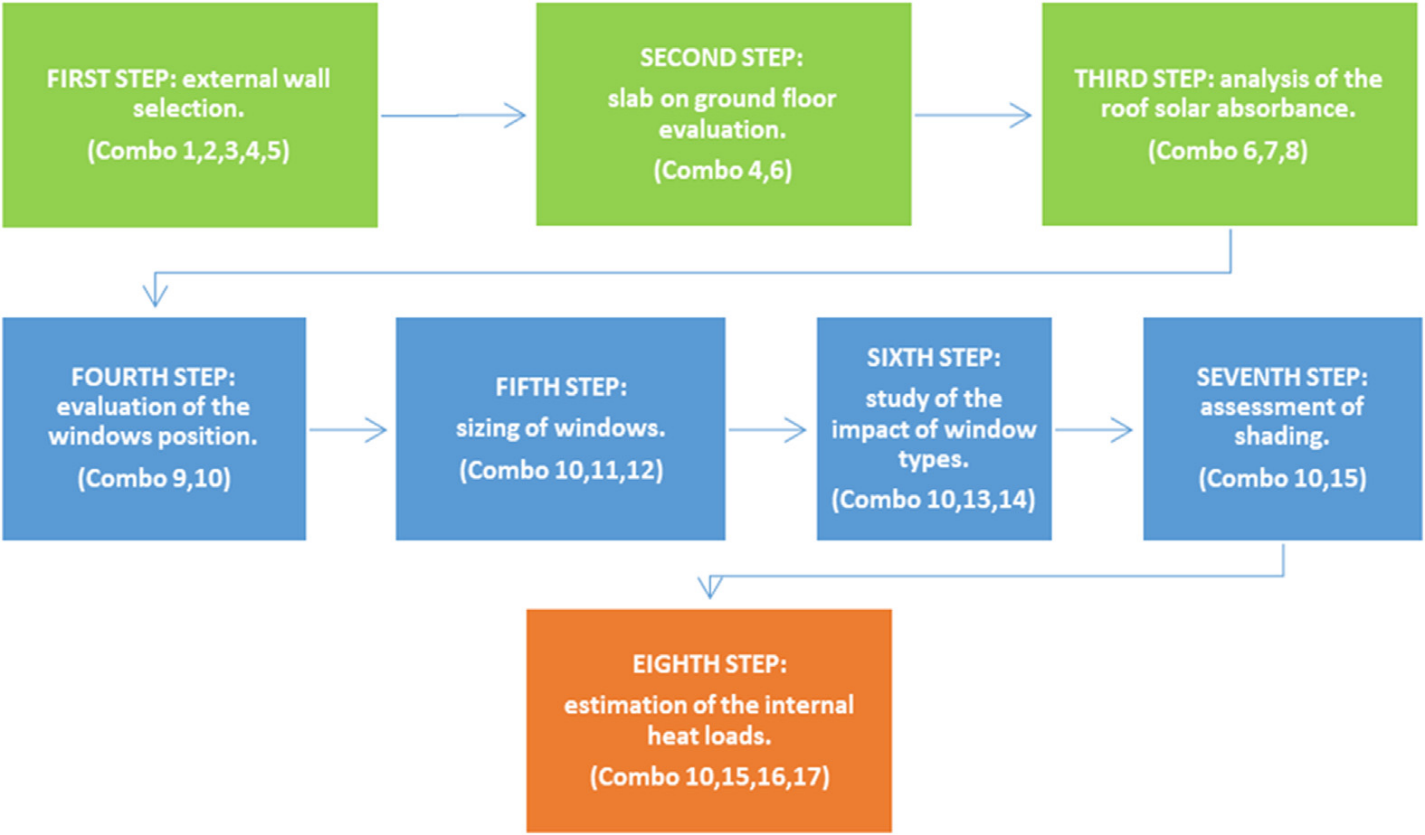
Development of the methodology.

A sequential search technique has been conducted in this study. Different building components are sequentially evaluated in order to investigate how each component impacts the thermal behaviour of the whole building. The development of the study has been shown in Fig. 2.

The software TRNSYS (Transient System Simulation) has been used to carry out the dynamics simulations. The case study has been realized through a special GUI program (TRNBuild), an add-on of TRNSYS. The methodology and all details of the input data of each single technical measure, are reported in [1].
